# Protein biomarkers for male artificial insemination subfertility in bovine spermatozoa

**DOI:** 10.1002/rmb2.12021

**Published:** 2017-03-20

**Authors:** Hiroshi Harayama, Kenta Minami, Kazumi Kishida, Taichi Noda

**Affiliations:** ^1^ Division of Animal Science Department of Bioresource Science Graduate School of Agricultural Science Kobe University Kobe Japan; ^2^ Department of Obstetrics and Gynecology Shiga University of Medical Science Otsu Japan; ^3^ Research Institute for Microbial Diseases Osaka University Suita Osaka Japan

**Keywords:** acrosome, artificial insemination, cattle, sperm, subfertility

## Abstract

**Background:**

Although artificial insemination (AI) technique is an established biotechnology for bovine reproduction, the results of AI (conception rates) have a tendency to decline gradually. To our annoyance, moreover, AI‐subfertile bulls have been occasionally found in the AI centers. To resolve these serious problems, it is necessary to control the sperm quality more strictly by the examinations of sperm molecules.

**Methods:**

We reviewed a number of recent articles regarding potentials of bovine sperm proteins as the biomarkers for bull AI‐subfertility and also showed our unpublished supplemental data on the bull AI‐subfertility associated proteins.

**Main findings:**

Bull AI‐subfertility is caused by the deficiency or dysfunctions of various molecules including regulatory proteins of ATP synthesis, acrosomal proteins, nuclear proteins, capacitation‐related proteins and seminal plasma proteins.

**Conclusion:**

In order to control the bovine sperm quality more strictly by the molecular examinations, it is necessary to select suitable sperm protein biomarkers for the male reproductive problems which happen in the AI centers.

## 1 Introduction

In mammals, including the human, male infertility and subfertility are due to defects in testicular spermatogenesis, epididymal sperm maturation, sperm transportation through the male reproductive tract, functions of the sperm molecules or other functions of the male reproductive organs. Males with one of these dysfunctions suffer from obstructive azoospermia, non‐obstructive azoospermia, oligozoospermia, varicocele, asthenospermia, absent vas deferens, pyospermia, retrograde ejaculation, erectile dysfunction, or other diseases.^1^, ^2^, ^3^, ^4^, ^5^ In the human, infertile men can undergo medical treatments with conventional and advanced biotechnologies, including artificial insemination (AI) by husband, conventional in vitro fertilization (IVF), intra‐cytoplasmic sperm injection (ICSI), and micro‐testicular sperm extraction.^6^, ^7^, ^8^, ^9^, ^10^, ^11^, ^12^ Henceforth, it might become important to argue that the use of novel findings and techniques (for instance, in vitro generation of male germ cells and gene therapy) in assisted reproductive technology should be used to overcome male infertility or subfertility.^13^, ^14^, ^15^, ^16^, ^17^

Meanwhile, the infertile or subfertile males in cattle rarely undergo these treatments, are not selected as sires for animal production, and are finally culled.^18^ However, there is a history that conventional biotechnologies were originally devised to improve the efficiency of bovine production in farms and subsequently they were applied to the clinical treatment of infertile humans. For example, it was discovered that glycerol works well as a cryoprotectant in the semen extender and cryopreservation techniques were established for bull spermatozoa.^19^ Since this breakthrough, the AI technique with cryopreserved spermatozoa has been used intensively for the production of bovine offspring in many countries and has made a large contribution to the industrial development of bovine production. In Japan, this reproductive technique is essential for the wide use of high‐performance sires and it has enabled the production of a large number of bovine offspring with excellent genetic traits. Moreover, it has allowed a decrease in the number of sires and the cutting of costs for the feeding and transportation of sires. For several decades, bovine reproduction generally has been conducted by using the AI technique with cryopreserved spermatozoa.

In cattle, high‐grade ejaculates (which are collected from the sires with excellent genetic traits and then which completely pass the examination of general characteristics [volume, color, and pH of the semen and concentration, motility, morphology, and acrosome integrity of the spermatozoa]) usually are used for the production of cryopreserved spermatozoa. In brief, the ejaculates are diluted with the extender and slowly cooled to 4‐5°C. Next, they are further diluted with the cold extender containing glycerol, pulled into the straws, rapidly frozen in the gas that evaporates from the liquid nitrogen, and then stocked in the liquid nitrogen. Dozens‐to‐several hundreds of semen straws are produced from one ejaculate. Before use for AI, several testing straws are rapidly thawed in warm water and frozen‐thawed spermatozoa are used for the motility assessment to examine the sperm's tolerance to cryopreservation (freezability). The straws with the same lot number as the testing straws that passed this final examination are preserved in the liquid nitrogen until use for AI. When a cow becomes in an optimum condition of the estrus cycle for AI, one of the cryopreserved straws is thawed and subsequently frozen‐thawed spermatozoa are injected into the uterine body of the cow. Thus, only the cryopreserved spermatozoa with excellent genetic traits and good motility are actually used for AI in cattle. However, the conception rates in bovine AI programs have been gradually declining in Japan and other countries for the last 20 years.^20^, ^21^ Moreover, there are large variations in the AI results (conception rates) among individual bulls^22^ and especially the AI‐subfertile bulls (males with low AI results) that are found occasionally in farms.^23^, ^24^, ^25^, ^26^ For the purpose of resolving these severe problems, various efforts have been made to determine the relationship between hormones and male reproductive traits and to predict the AI fertility of bulls by the examination of sperm characteristics.^18^, ^27^ For instance, many previous reports^26^, ^28^, ^29^, ^30^, ^31^ showed that conventional examinations of the plasma membrane, motility, morphology, and acrosome in cryopreserved spermatozoa could be fairly contributive to the evaluation of sperm quality and prediction of bull AI fertility. In addition, there is an interesting report that implies that bull spermatozoa with a morphologically abnormal head are less capable of swimming up to the ampulla of the oviduct than morphologically normal spermatozoa, but that the spermatozoa with vacuoles in the head can reach oocytes in vivo, as well as morphologically normal spermatozoa.^32^ For examinations of the sperm genome for the prediction of bull AI fertility, the sperm chromatin structure assay with acridine orange staining,^33^, ^34^ evaluation of DNA damage by the terminal deoxynucleotidyl transferase‐mediated dUTP nick end labeling (TUNEL) assay,^35^ and sperm DNA methylation analyses^36^ are available for cattle. Moreover, one of the well‐working assays is the superovulation/AI embryo‐collection test, which was designed for Japanese Black cattle by Fukushima et al. Detailed methods of this test are shown in the authors’ previous report.^26^ However, it is necessary to devise new examinations of sperm molecular characteristics in order to evaluate bull AI fertility exactly in a short time. In this review, the potential of bovine sperm proteins as the biomarkers for bull AI subfertility is described. Moreover, unpublished supplemental data also are shown on the bull AI subfertility‐associated proteins that are currently under the authors’ investigation.

## 2 Sperm Proteomics

In order to screen for the protein biomarkers of male AI fertility, detergent‐extracted proteins of bovine spermatozoa were compared by proteomic analysis between bulls with different AI fertility rates. A study showed the proteome profiles of spermatozoa from high‐AI‐fertile (3569 kinds of proteins) and low‐AI‐fertile (3799 kinds of proteins) bulls (Holstein) and reported that 51 and 74 sperm proteins were included more largely in the spermatozoa from the bulls with higher and lower AI fertility rates, respectively.^37^ Further analyses that used GO slim and ingenuity pathway analysis indicated that the former proteins were probably functional in energy metabolism, cellular movement, cellular interaction, the cell cycle, or spermatogenesis and that the latter proteins might be involved in cell death or reproductive system disease. Specifically, the epidermal growth factor signaling cascade, platelet‐derived growth factor signaling cascade, oxidative phosphorylation pathway, and pyruvate metabolism pathway were prominent in the spermatozoa from the bulls with higher AI fertility rates. In the spermatozoa from the bulls with lower AI fertility rates, the signaling cascades for the cell cycle (G2/M) DNA damage check point regulation and apoptosis tended to be more functional.^37^

In another proteomic analysis, adenylate kinase isoenzyme 1 (AK1) and phosphatidylethanolamine‐binding protein 1 (PEBP1) were detected abundantly in the spermatozoa from the bulls (Holstein) with higher AI fertility rates.^38^ In contrast, the T‐complex protein 1 subunits ε and θ (CCT5 and CCT8), epididymal sperm‐binding protein E12 (ELSPBP1), proteasome subunit α type‐6, and binder of sperm 1 (BSP1) were predominately abundant in the spermatozoa from the bulls with lower AI fertility rates. The results regarding AK1, PEBP1, ELSPBP1, and BSP1 were confirmed by Western blotting analyses. Moreover, the relationship between the AI fertility rates and the abundance of these proteins was analyzed by using the linear regression model. This model established that CCT5 and AK1 explained a significant proportion of the variation in the AI fertility rates.

The third report showed that five proteins (enolase 1 [ENO1, α‐ENO], adenosine triphosphate [ATP] synthase H^+^ transporting mitochondrial F1 complex β subunit, apoptosis‐stimulating of p53 protein 2, α‐2‐HS‐glycoprotein, and phospholipid hydroperoxide glutathione peroxide) and three proteins (voltage‐dependent anion channel 2 [VDAC2], ropporin‐1, and ubiquinol‐cytochrome‐c reductase complex core protein 2 [UQCRC2]) were more largely included in the spermatozoa from the bulls (Hanwoo; Korean native cattle) with higher and lower AI fertility rates, respectively.^39^ Among these proteins, ENO1, VDAC2, and UQCRC2 were significantly correlated with individual AI fertility rates. According to the another report,^40^ moreover, sperm ENO1 was down‐regulated in the lower‐AI‐fertile bulls.

All of these reports suggest that ATP synthesis‐related molecules are more largely included in the spermatozoa from bulls with higher AI fertility rates. This suggestion is supported by the observation that the measurement of total ATP formation in bull cryopreserved spermatozoa has been positively correlated with the results of AI.^41^ These indicate that the lower activity of the sperm pathways for ATP synthesis is one of the causal factors of bull AI subfertility. The ATP is indispensable for the initiation, regulation, and maintenance of the progressive motility of bull spermatozoa, which is required for successful AI.^42^, ^43^, ^44^, ^45^, ^46^ Thus, it is important to conduct an objective investigation of the motility of bull cryopreserved spermatozoa before use in AI with a microscopic image recorder or computer‐associated sperm analyzer.^30^, ^31^, ^47^

## 3 Acrosomal Proteins

The mammalian spermatozoon is composed of a head and a flagellum that are connected with each other at the neck.^48^ As mentioned above, bull AI subfertility that is caused by dysfunctions of ATP synthesis‐related pathways in the sperm flagellum is probably detectable by an objective examination of the progressive motility of the cryopreserved spermatozoa. However, the authors’ question is why AI‐subfertile bulls are found occasionally on farms where only cryopreserved spermatozoa with good motility are used for AI. Thus, the focus has been on the examination of the acrosomal proteins of the sperm head.

The sperm head is divided into the acrosomal and postacrosomal regions; furthermore, the acrosomal region is subdivided into the marginal, principal, and equatorial segments.^48^ The equatorial segment contains a unique compartment called the “equatorial subsegment.”^49^ The bovine spermatozoon with the spatulate shape (like a Japanese rigid fan) possesses a relatively larger acrosomal principal segment and a smaller equatorial segment, compared with the mouse spermatozoon with the falciform (falx‐like) shape.^48^ However, the triple‐membrane structures (plasma, outer‐acrosomal, and inner‐acrosomal membranes) and protein components in the acrosomal regions are conserved among many species of mammals.^48^, ^50^ In this section, the authors’ data regarding bovine sperm acrosomal proteins, which are potentially bull AI‐subfertility biomarkers, are shown.

### 3.1 Acrosomal tyrosine‐phosphorylated proteins

**Figure 1:**
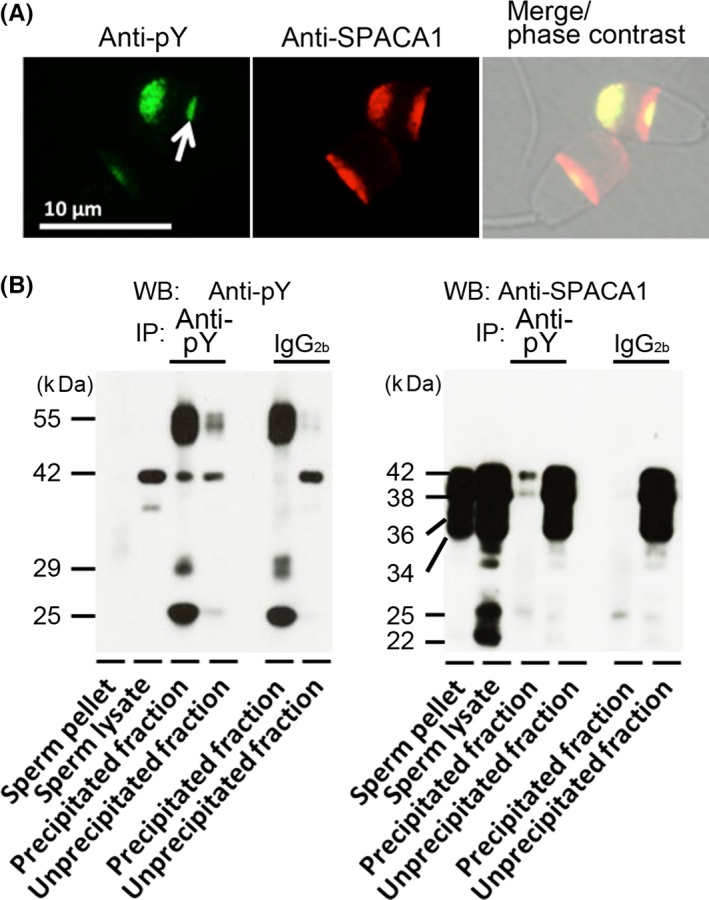
Immunodetection of tyrosine‐phosphorylated proteins and sperm acrosome‐associated 1 (SPACA1) proteins in the spermatozoa that were collected from Japanese Black bulls. A, Tyrosine‐phosphorylated proteins (green fluorescence) and SPACA1 proteins (red fluorescence) were detected in the cauda epididymal spermatozoa by double immunostaining (a representative of six replicates). The cauda epididymal spermatozoa were washed with a phosphate‐buffered saline that contained polyvinyl alcohol (PBS‐PVA) three times, treated with mouse anti‐phosphotyrosine (pY) monoclonal antibody or guinea pig anti‐SPACA1 polyclonal antibody, and subsequently with fluorescein isothiocyanate‐conjugated or tetramethylrhodamine‐conjugated secondary antibodies, as described previously.^25^, ^56^ An arrow indicates the distribution of the acrosomal tyrosine‐phosphorylated proteins in the equatorial subsegment. B, A tyrosine‐phosphorylated form of the SPACA1 proteins in the lysates from the ejaculated spermatozoa was detected by immunoprecipitation (IP)–Western blotting (WB) (a representative of three replicates). The ejaculated spermatozoa were washed with PBS‐PVA three times and then used for the IP‐WB, as described previously.^25^ Ig, immunoglobulin

**Figure 2:**
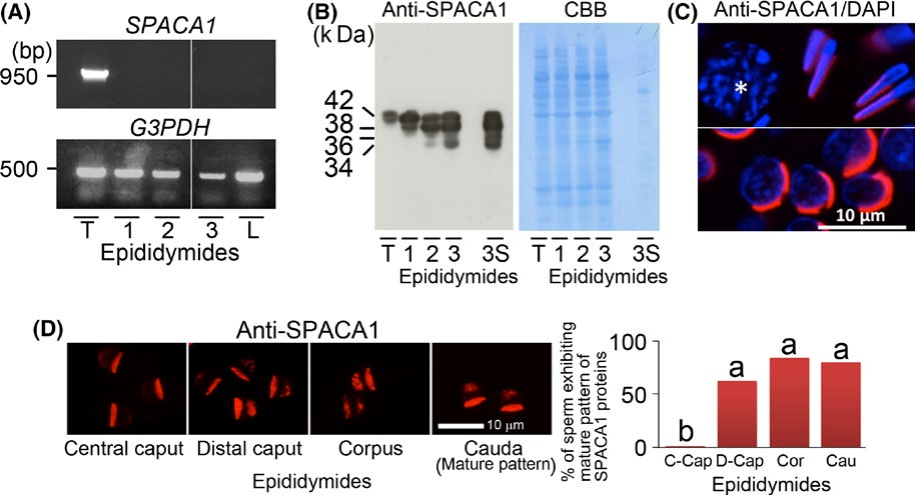
Detection of sperm acrosome‐associated 1 (SPACA1) proteins in the testes, epididymides, and spermatozoa that were collected from Japanese Black bulls. A, Bull *SPACA1* messenger RNA expression was examined in the testes (T), epididymides (1, central caput; 2, distal caput; 3, cauda), and livers (L) by reverse‐transcription polymerase chain reaction (PCR), as described previously^62^ (a representative of three replicates). The PCR products were separated in agarose gel containing ethidium bromide. Glycerol‐3‐phosphate dehydrogenase (*G3PDH*) was amplified as the control in every experiment. B, The SPACA1 proteins were detected in the testes (T, testicular tissue extracts), epididymides (1, central caput tissue extracts; 2, distal caput tissue extracts; 3, cauda tissue extracts, and 3S, cauda epididymal sperm extracts) by Western blotting (WB) (a representative of three replicates), as described previously.^25^, ^62^ After the WB, each membrane was stained with Coomassie Brilliant Blue G‐250 (CBB). C, The SPACA1 proteins were detected in the frozen sections of the paraformaldehyde‐fixed testes by indirect immunofluorescence (a representative of three replicates). After immunostaining with the anti‐SPACA1 protein antibody and tetramethylrhodamine‐conjugated secondary antibody (red fluorescence), each preparation was counterstained with 4′,6‐diamidino‐2‐phenylindole (blue fluorescence). The SPACA1 proteins were detected in the early spermatids in the lower photograph and in the late spermatids in the upper photograph, but not in the spermatocyte with the asterisk in the upper photograph. D, The distribution patterns of the SPACA1 proteins were observed in the immunostained epididymal spermatozoa^25^, ^62^ and the percentages of the spermatozoa that exhibited a mature (normal distribution) pattern of SPACA1 proteins were calculated, as described.^55^ There were significant differences between the values with different letters (*P*<.05). C‐Cap, central caput; D‐Cap, distal caput; Cor, corpus; Cau, cauda

The tyrosine‐phosphorylated proteins of the sperm head of bulls (Japanese Black cattle) are localized mainly in the acrosomal principal segment and equatorial subsegment.^25^ One of the tyrosine‐phosphorylated proteins is a sperm acrosome‐associated 1 (SPACA1) protein in boar spermatozoa.^51^, ^52^ This also was observed in bull (Japanese Black cattle) spermatozoa by the double‐staining (upper sperm head of Fig. 1A) and immunoprecipitation–Western blotting (Fig. 1B) with anti‐phosphotyrosine (anti‐pY) antibody and anti‐SPACA1 protein antibody. The SPACA1 proteins originally were found as human sperm antigens, the “sperm acrosomal membrane‐associated protein 32”, with molecular masses from 32 to 34 kDa that were produced restrictedly in the testes.^53^ In the mouse testis, the SPACA1 proteins were shown to play indispensable roles in the acrosomal formation of spermatids during spermiogenesis.^54^ The authors showed that SPACA1 proteins were produced in bull (Japanese Black cattle) testes (Fig. 2A,B) and localized in the acrosomal part of the bull spermatids (Fig. 2C), suggesting that the bull SPACA1 proteins could have the same functions in spermiogenesis as the mouse SPACA1 proteins. Moreover, the authors observed that the bull SPACA1 proteins exhibited changes in their molecular masses and were translocated to the acrosomal principal segment during sperm transit through the epididymis (Fig. 2D). Likewise, the tyrosine‐phosphorylated proteins were translocated to the acrosomal principal segment during bull sperm maturation.^55^

In the head of bull (Japanese Black cattle) mature spermatozoa, the tyrosine‐phosphorylated proteins usually were distributed in the acrosomal principal segment and the equatorial subsegment (upper sperm head of Fig. 1A, normal distribution [mature pattern]) and at least an aliquot of them was a 42 kDa tyrosine‐phosphorylated SPACA1 protein (Fig. 1B). However, some of the bull spermatozoa apparently were lacking in these proteins of the acrosomal principal segment (lower sperm head of Fig. 1A, abnormal distribution [immature pattern]^25^). In the authors’ investigation of 20 bulls,^55^ their ejaculates had normal general characteristics of spermatozoa (progressive motility, morphological normality, and acrosome integrity) but they showed large individual variations in the percentages of spermatozoa with a normal distribution pattern (mature pattern) of the acrosomal tyrosine‐phosphorylated proteins of between 4% and 99%. Similar results were obtained for the cauda epididymal spermatozoa and cryopreserved spermatozoa. Moreover, these indices on the acrosomal phosphoproteins of the epididymal, ejaculated, and cryopreserved spermatozoa were positively correlated with the conception rates in AI and the percentages of cryopreserved spermatozoa with morphologically normal acrosomes.^26^, ^55^ These results suggest that a lack of the acrosomal phosphoproteins (one of the main components is probably the 42 kDa tyrosine‐phosphorylated SPACA1 protein) in ejaculated spermatozoa with normal general characteristics is linked to bull AI subfertility and lower sperm freezability. However, molecular analyses of these proteins are required for disclosing how the SPACA1 proteins can protect the acrosomes during the cryopreservation process and consequently maintain their sperm‐fertilizing ability.

According to investigations of human spermatozoa from infertile patients,^56^ human SPACA1 proteins might not be tyrosine‐phosphorylated, unlike bovine SPACA1 proteins. However, their distribution patterns in the acrosomal principal segment are varied among human spermatozoa, as with bovine SPACA1 proteins, and could be classified into three grades. In the previous experiment, the surplus of sperm samples that were prepared for the clinical treatments of conventional IVF were used for the immunostaining of the SPACA1 proteins. The spermatozoa were graded according to the distribution pattern of the SPACA1 proteins and the obtained SPACA1 indices were largely varied among the patients by between 13 and 199 points (full marks: 200 points). These indices were highly correlated with the results of conventional IVF (developmental rates of embryos to blastocysts), suggesting that the SPACA1 indices could be valid as biomarkers that can predict the effectiveness of conventional IVF for human infertile patients.

### 3.2 IZUMO1

An acrosomal protein, “IZUMO1,”^57^, ^58^, ^59^ was discovered in mouse spermatozoa as an essential mediator of the interaction and fusion with oocytes. This sperm‐specific transmembrane protein possesses the IZUMO‐specific domain and immunoglobulin domain. Further observations showed that this protein is originally distributed in the acrosomal principal segment of mature spermatozoa, is translocated to the surface of the equatorial segment during the acrosome reaction,^60^ and that subsequently the IZUMO1 of the equatorial segment binds to its receptor “JUNO” of the oocyte plasma membrane in the perivitelline space and mediates sperm fusion with the oocyte plasma membrane.^61^ In the bovine testes (Japanese Black cattle), an ortholog of mouse *Izumo1* gene was expressed.^62^ Its translation product (a 52 kDa precursor form) was localized along the border between the acrosomal principal and equatorial segments and underwent maturation‐related changes to a 45 kDa form during sperm transit through the epididymis. Moreover, multiple staining with anti‐IZUMO1 antibody and fluorescein isothiocyanate–peanut agglutinin revealed that bovine IZUMO1 was localized along the border between the acrosomal principal and equatorial segments, not merely in freshly ejaculated spermatozoa (spermatozoa with intact acrosomes) but also in the spermatozoa that were undergoing the acrosome reaction (spermatozoa with severely damaged acrosomes), and that it became detectable in the equatorial segment in the spermatozoa after the acrosome reaction (spermatozoa without acrosomes). This suggests that the accomplishment of the acrosome reaction might be necessary for the translocation of bovine IZUMO1 to the equatorial segment.

The authors also investigated the acrosome morphology and distribution pattern of IZUMO1 in ejaculated spermatozoa from 10 bulls (Japanese Black cattle) by multiple staining and found that most of the spermatozoa possessed a normal acrosome and showed the normal distribution pattern of IZUMO1 (along the border between the acrosomal principal and equatorial segments) in almost all of the samples.^62^ Namely, there was no large individual difference in the distribution patterns of IZUMO1 in the ejaculated spermatozoa among bulls. Thus, bovine IZUMO1 is unlikely to be a valid biomarker of bull AI subfertility. However, approximately half of the cryopreserved spermatozoa with severely damaged acrosomes still showed the normal distribution pattern of IZUMO1 and the others possessed IZUMO1 in the equatorial segment (like acrosome‐reacted spermatozoa) or lost IZUMO1. These observations are interpreted as showing the occurrence of aberrant translocation and the loss of bovine IZUMO1 in many spermatozoa during the process of cryopreservation. It is speculated that these defective behaviors of bovine IZUMO1 might reduce the conception rates in the AI program.

## 4 Nuclear Proteins

Mammalian spermatozoa have a mission of transporting the paternal haploid genome to the oocytes. For the purpose of protecting the sperm genome from damage before fertilization, sperm chromatins are hypercondensed. This hypercondensation is made in the process of spermiogenesis, during which haploid spermatids are elongated and transformed to the spermatozoa in the seminiferous tubules. In the nuclei, most of the core histones are substituted transiently by the transition proteins and finally by the protamines.^63^ Many reviews previously have described the details of the relationship between male infertility and epigenetics at the histones and protamines of male germ cells.^64^, ^65^, ^66^, ^67^, ^68^ In this section, information is introduced on bovine sperm protamines and their relationship with bull AI subfertility.

### 4.1 Protamines

Sperm nuclei contain at least two forms of protamines that are different among species. Protamine 1 is universally detectable in the mammalian spermatozoa, while protamine 2 is present in certain placental mammals, including primates and rodents.^69^ As bull spermatozoa include only protamine 1, the sperm DNA protamination state can be compared among bulls with different AI fertility rates by immunodetection of protamine 1 and by the toluidine blue staining of the sperm's remaining histones. Moreover, the DNA fragmentation that is associated with histone–protamine transition errors also is detectable by sperm chromatin dispersion tests. In examinations with these techniques, one of those showed that the spermatozoa from bulls with lower AI fertility rates showed inadequate chromatin protamination and DNA disintegration at higher rates and suggested that the defects in the sperm chromatin condensation is associated with the reduction of AI fertility rates.^70^ Moreover, another study showed that the contents of sperm protamines were related closely to the occurrence of sperm DNA damage in bulls (Indian cattle) and indicated that protamine deficiency might cause the instability and damage of sperm DNA, leading to the reduction of bull AI fertility rates.^71^ Thus, the detection of spermatozoa with a lack of protamines is indicative of bull AI subfertility. The research regarding Nelore cattle showed that the spermatozoa from young bulls had larger head diameters due to the lower protamination level and resultant deficiency of chromatin condensation, compared with the spermatozoa from adult bulls.^72^ This might account for the cause of unstable results of AI when using cryopreserved spermatozoa from young bulls.

## 5 Capacitation‐related Proteins

Immediately after ejaculation, mammalian spermatozoa cannot penetrate into the oocytes. They become capable of fertilizing oocytes during their stay in the site‐specific environment that is produced by the female reproductive tract.^73^, ^74^ This process is termed “capacitation” and was discovered by Chang^75^ and Austin.^76^, ^77^ Capacitation includes a variety of physiological changes on the sperm surface and in the intracellular space. Specifically, the sperm plasma membrane becomes unstable from the release of decapacitation factors during the early stage of capacitation.^78^, ^79^ The intracellular signal transduction systems gradually become active and promote the increase of pH_*i*_, protein phosphorylation, and membrane hyperpolarization during both the early and the late stages in order to induce the entry of the external Ca^2+^ and release of the internal Ca^2+^ from the store.^80^, ^81^ In the case of considering Chang's meaning of capacitation, moreover, it is preferable to include the Ca^2+^‐triggered events of hyperactivation and acrosome reaction in the capacitation process.^82^

According to a previous article on a certain breed of beef bull (Red Angus cattle),^83^ male infertility might be caused by a failure to complete the sperm capacitation process. It has been believed for a long time that the progress of the capacitation process is coincident with the increase of tyrosine‐phosphorylated proteins in mammals, including cattle,^84^, ^85^, ^86^ although the detailed functions of the tyrosine‐phosphorylated proteins are poorly understood in the fertilization‐related events of bull spermatozoa.^87^ This event is regulated pivotally by the intracellular cyclic adenosine monophosphate (cAMP) signaling cascades that are composed of the bicarbonate/Ca^2+^‐activated adenylyl cyclase (a soluble‐type of adenylyl cyclase, ADCY10), protein kinase A (PKA), protein phosphatases, and protein tyrosine kinases.^81^, ^88^, ^89^, ^90^ Moreover, various ion channels and pumps play regulatory roles in the multiple changes during capacitation.^91^, ^92^, ^93^ Thus, a number of sperm functional proteins are involved in the regulation of the capacitation process. In this section, information is introduced on the relationship of the capacitation‐related proteins with bull AI subfertility.

### 5.1 ADCY10

A soluble type of adenylyl cyclase, “ADCY10,”^94^, ^95^, ^96^, ^97^, ^98^ is unique in its lack of transmembrane domains, independence of G‐protein‐coupled receptors, and direct stimulation by the interaction with bicarbonate and Ca^2+^, compared with the transmembrane types of the adenylyl cyclase, “ADCY1‐9.” This isoform is synthesized most abundantly in mouse testes as either the 189 kDa full‐length form or the 48 kDa truncated form from the same *Adcy10* gene by alternative splicing. Although both forms contain two cyclase domains that catalyze the conversion from ATP to cAMP, the specific activity of the truncated form is 20‐fold higher than that of the full‐length form. Moreover, manipulation of this gene showed that this enzyme is indispensable in the progress of the capacitation process and subsequent successful fertilization. For bulls (Japanese Black cattle), the authors showed that ADCY10 was distributed in the neck and flagellar principal piece of the ejaculated spermatozoa and that both messenger RNAs (mRNAs) that coded the full‐length and truncated forms were expressed in the testes by the alternative splicing of exon 11.^99^ Interestingly, it also was found that the splicing error yielded the other variant of *ADCY10*, with the aberrance in the second cyclase domain by retaining the intronic nucleotides (four bases, CCAG) that connect to the initial part of exon 10, and that the incidence rates of this splicing error were largely varied among individual bulls by between 0% and 54.5%. The authors are concerned that such splicing errors in mRNA coding in the important capacitation‐regulatory protein might cause bull AI subfertility.

### 5.2 Na^+^/K^+^‐ATPase

A model has been proposed for the induction of bull sperm capacitation by the interaction between Na^+^/K^+^‐ATPase and its inhibitor, “ouabain.”^100^, ^101^ It is likely that the inhibition of the Na^+^/K^+^‐ATPase activity with ouabain promotes an increase of the intracellular Ca^2+^ (by the suppression of the Na^+^/Ca^2+^ exchanger activity), PKA activation, and tyrosine phosphorylation, leading to capacitation. Moreover, the interaction between Na^+^/K^+^‐ATPase and ouabain might activate the extracellular signal‐regulated kinases 1/2, phospholipase C, protein kinase C and protein tyrosine kinase signaling cascades, leading to capacitation. This ion transporter, Na^+^/K^+^‐ATPase,^100^, ^102^ is generated in the testes and subsequently is localized in the head (postacrosomal region) and middle piece of the ejaculated spermatozoa. In the experiment in which the testicular temperature was elevated in bulls (Holstein) by scrotal insulation,^101^ its generation was awfully disturbed in the testes and its content was largely decreased in the spermatozoa. Although the deterioration of the general characteristics (motility and morphology) of the ejaculated spermatozoa also was observed in the experiment of scrotal insulation, a moderate decrease in the sperm contents of Na^+^/K^+^‐ATPase might cause precocious capacitation in the spermatozoa with normal motility and morphology. Moreover, there were individual variations in the Na^+^/K^+^‐ATPase activity of the frozen spermatozoa among beef bulls.^101^ It is expected that a lower level of activity of Na^+^/K^+^‐ATPase is potentially a biomarker for bull AI subfertility, owing to the abnormal process of sperm capacitation and the consequent failure of fertilization.

## 6 Seminal Plasma Proteins

At ejaculation, the cauda epididymal spermatozoa are mixed with the secretory fluids from the accessary genital glands, including the seminal vesicles, prostate, urethral glands and bulbourethral glands. In the semen that is collected for cryopreservation, the spermatozoa are swimming in the seminal plasma, which contains a variety of proteins that is secreted from the epididymides and accessary genital glands and that can minimize cryoinjury effects on the sperm viability and motility and acrosome integrity.^103^ In this section, information is introduced regarding the fertility‐associated proteins.

### 6.1 Fertility‐associated proteins

One study discovered the presence of four kinds of fertility‐associated proteins in bull (Holstein) seminal plasma.^104^ Two‐dimensional electrophoresis showed that osteopontin (55 kDa, pI=4.5)^105^, ^106^ and lipocalin‐type prostaglandin D synthase (26 kDa, pI=6.2)^107^ were more abundantly included in the seminal plasma from bulls with higher AI fertility rates. The osteopontin (Ca^2+^‐binding protein) of the seminal plasma originates from the epithelial cells of the seminal vesicle and ampulla.^106^ This protein also was detectable in the cauda epididymal fluid and the testicular parenchyma homogenates as the 55 kDa form and the 25 kDa form, respectively, and was localized as the 35 kDa form in the postacrosomal region and middle piece of the ejaculated spermatozoa.^108^ In contrast, the lipocalin‐type prostaglandin D synthase was detectable in the elongating spermatids and Sertoli cells of the seminiferous tubules, cuboidal epithelial cells of the rete testis and efferent ducts, and the epithelial principal cells of the epididymides and was localized in the apical ridge of the acrosome on the ejaculated spermatozoa.^109^ In addition, the seminal plasma from the bulls with lower AI fertility rates more prominently included two other proteins (16 kDa, pI=4.1 and 16 kDa, pI=6.7),^104^ which might be biomarkers for bull AI subfertility.

## 7 Conclusion

**Figure 3:**
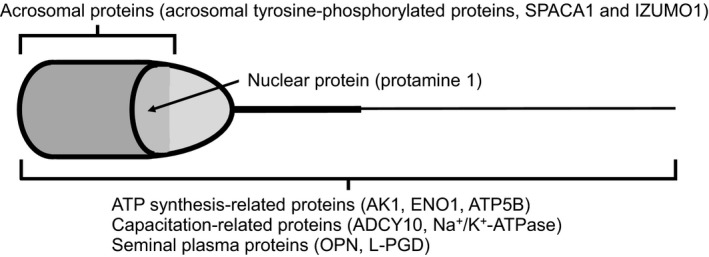
Bovine sperm proteins that are potentially associated with bull artificial insemination (AI) subfertility because of a deficiency or dysfunction of these sperm proteins. ADCY10, adenylyl cyclase 10; AK1, adenylate kinase 1; ATP, adenosine triphosphate; ATP5B, ATP synthase H^+^ transporting mitochondrial F1 complex β subunit; ENO1, enolase 1; L‐PGD, lipocalin‐type prostaglandin D synthase; OPN, osteopontin; SPACA1, sperm acrosome‐associated 1

For the purpose of finding out those AI subfertile bulls exactly in a short time, it is necessary to evaluate not merely the general characteristics (progressive motility, morphological normality, and acrosome integrity) but also the molecular characteristics of the protein biomarkers in freshly ejaculated and cryopreserved spermatozoa. Actually, the authors and their colleagues have been investigating the general characteristics and distribution patterns of acrosomal tyrosine‐phosphorylated proteins (a protein biomarker for bull AI subfertility) in freshly ejaculated and cryopreserved spermatozoa from most of the sire candidates in the bovine AI center of Hyogo Prefecture, Japan.^26^, ^55^ However, it should be noted that a key point is to select suitable protein biomarkers for the sperm problems that happen in the AI centers because bull AI subfertility is caused by a deficiency or dysfunction of various molecules, including the regulatory proteins of ATP synthesis, acrosomal proteins, nuclear proteins, capacitation‐related proteins, and seminal plasma proteins (Fig. 3). In addition, it is emphasized to the readers in the medical field that SPACA1 indices are valid as biomarkers that can predict the effectiveness of conventional IVF for human infertile patients.

## Disclosures

*Conflict of interest*: The authors declare no conflict of interest. *Human studies*: This article does not contain any study with human participants that was performed by any of the authors. *Animal studies*: All the institutional and national guidelines for the care and use of animals were followed.
